# Notch and MAML-1 Complexation Do Not Detectably Alter the DNA Binding Specificity of the Transcription Factor CSL

**DOI:** 10.1371/journal.pone.0015034

**Published:** 2010-11-24

**Authors:** Cristina Del Bianco, Anastasia Vedenko, Sung Hee Choi, Michael F. Berger, Leila Shokri, Martha L. Bulyk, Stephen C. Blacklow

**Affiliations:** 1 Department of Biological Chemistry and Molecular Pharmacology and Department of Pathology, Harvard Medical School, Boston, Massachusetts, United States of America; 2 Department of Medicine, Harvard Medical School and Brigham and Women's Hospital, Boston, Massachusetts, United States of America; 3 Department of Pathology, Harvard Medical School and Brigham and Women's Hospital, Boston, Massachusetts, United States of America; 4 Committee on Higher Degrees in Biophysics, Harvard University, Cambridge, Massachusetts, United States of America; 5 Division of Health Sciences and Technology, Harvard Medical School, Boston, Massachusetts, United States of America; 6 Department of Cancer Biology, Dana Farber Cancer Institute, Boston, Massachusetts, United States of America; University of Texas MD Anderson Cancer Center, United States of America

## Abstract

**Background:**

Canonical Notch signaling is initiated when ligand binding induces proteolytic release of the intracellular part of Notch (ICN) from the cell membrane. ICN then travels into the nucleus where it drives the assembly of a transcriptional activation complex containing the DNA-binding transcription factor CSL, ICN, and a specialized co-activator of the Mastermind family. A consensus DNA binding site motif for the CSL protein was previously defined using selection-based methods, but whether subsequent association of Notch and Mastermind-like proteins affects the DNA binding preferences of CSL has not previously been examined.

**Principal Findings:**

Here, we utilized protein-binding microarrays (PBMs) to compare the binding site preferences of isolated CSL with the preferred binding sites of CSL when bound to the CSL-binding domains of all four different human Notch receptors. Measurements were taken both in the absence and in the presence of Mastermind-like-1 (MAML1). Our data show no detectable difference in the DNA binding site preferences of CSL before and after loading of Notch and MAML1 proteins.

**Conclusions/Significance:**

These findings support the conclusion that accrual of Notch and MAML1 promote transcriptional activation without dramatically altering the preferred sites of DNA binding, and illustrate the potential of PBMs to analyze the binding site preferences of multiprotein-DNA complexes.

## Introduction

CSL (gene name *RBPJ*) is a DNA-binding transcription factor that orchestrates the transcriptional response to Notch receptor activation. After ligand binding induces proteolysis of Notch, ICN migrates to the nucleus, where it binds to CSL and recruits a protein of the Mastermind family to upregulate transcription of a typical target gene [1], [2], [3]. In some contexts, CSL may act as a transcriptional repressor in the absence of activated Notch, either by recruiting histone modifying enzymes or by direct interaction with other corepressors [4], [5], [6]. Formation of a complex between Notch and CSL creates a binding groove that captures a Mastermind protein [7], [8], which in turn is thought to recruit generalized transcriptional coactivators such as p300/CBP and the basal transcription machinery to induce target gene expression [9], [10], [11].

The interaction of Notch with CSL is a thus crucial step in the signaling pathway because the loading of CSL onto DNA dictates which genes are transcribed in response to assembly of Notch-CSL-MAML complexes. The preferred DNA binding sites for murine CSL and for the protein LAG-1, which is the homologue of CSL in *C. elegans*, have been analyzed by electrophoretic mobility shift assay, selection-based methods, and a bacterial one-hybrid system. These methods led to the identification of an eight base-pair consensus binding sequence of CGTGGGAA [12].

More recently, a quantitative thermodynamic analysis of the interaction between CSL and the two individual CSL binding sites in the *HES-1* promoter have revealed subtle differences in the binding affinities of CSL for each DNA binding site [13]. Studies investigating the loading of Suppressor-of-hairless (Su(H), the *Drosophila* CSL homologue) onto DNA in cultured fly cells have shown that the occupancy of DNA binding sites by CSL increases after a pulse of Notch activation [14], [15]. However, it is not yet clear whether this change results from increased stability of DNA-bound complexes or alterations in binding site preferences, as none of these studies have directly investigated whether the interactions with Notch and Mastermind-like proteins might alter the DNA binding site preferences of CSL and/or its binding affinities for different DNA sequences.

Here we present a new strategy to analyze the binding of multiprotein CSL complexes to DNA. We exploited the universal protein binding microarray (PBM) technology [16], [17], [18], [19] to analyze and compare the DNA binding preferences of isolated CSL for DNA with the site preferences for multiprotein Notch-CSL and Mastermind-Notch-CSL complexes. These studies show that the binding of Notch and the subsequent recruitment of Mastermind-like-1 (MAML-1) do not detectably change the binding specificities of CSL, supporting the idea that the formation of Notch transcriptional activation complexes rely primarily on the binding of DNA by CSL to dictate target-site specificity.

## Results

In this study, we used PBMs to analyze the DNA-binding site preferences of CSL alone and in complex with the four different Notch receptors and human MAML-1. Each custom-designed, universal spot on the 4×44K 60-mer oligonucleotide array contains 26 distinct, overlapping 10-mers, resulting in not only complete but also highly redundant coverage of all 8-bp sequences: within each individual chamber containing approximately 44,000 60-mers, there are more than 1.1 million 10-mers displayed, and each possible 8-mer DNA sequence variant is present in either orientation at 32 spots. For each 8-mer, we calculate its median signal intensity over the 32 spots at which it is present and also a rank-based, PBM enrichment score (E-score), ranging from −0.5 (worst) to +0.5 (best), that indicates the preference of a protein or protein complex for that 8-mer [20]. Prior comparisons between PBM signal intensities and dissociation constant data for several eukaryotic proteins indicate that relative K_d_ values are approximately inversely correlated with the median signal intensity of each *k*-mer analyzed, indicating that relative signal intensities estimate DNA binding preferences [20].

**Figure 1:**
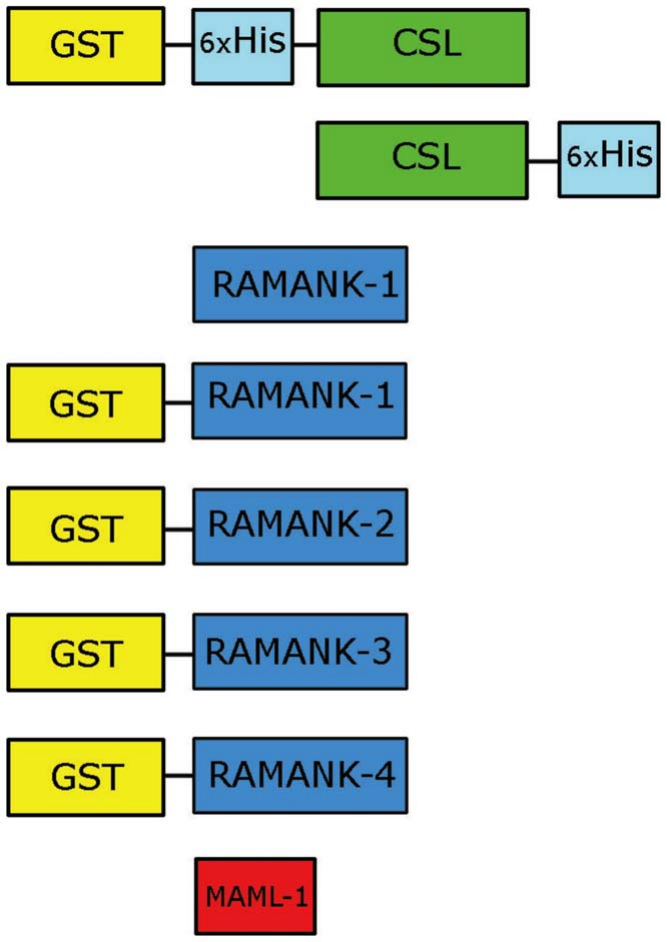
Schematic illustrating the protein constructs used in this study. Abbreviations are: RAMANK, a Notch polypeptide including the RAM and ankyrin repeat domains of Notch; MAML-1, Mastermind-like-1; GST, glutathione S-transferase; 6×His, hexahistidine tag.

To identify bound complexes, either the CSL protein, or the RAMANK portion of the Notch protein (hereafter referred to as Notch), was prepared as a glutathione-S-transferase (GST) fusion, which was detected with an anti-GST antibody coupled to an Alexa-488 fluorophore. The different CSL, Notch, and MAML-1 proteins tested in these studies are illustrated in Figure 1.

**Figure 2:**
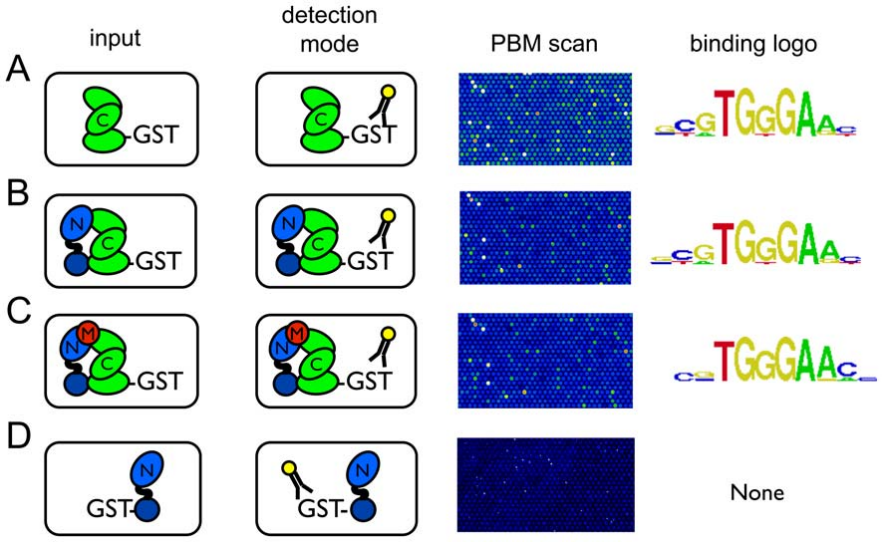
Accrual of Notch1 and MAML-1 does not detectably alter the binding site preferences of GST-CSL. Protein mixtures were applied to PBMs, and bound complexes were detected with an Alexa488-conjugated anti-GST antibody (see Materials and Methods). Columns from left to right show: input components, strategy used to detect immobilized proteins, zoom-in on portions of microarray images, and a DNA sequence logo representing the bound 8-mers [20]. The protein mixtures incubated in separate chambers of the same microarray chip were as follows: **A**) GST-CSL alone, **B**) GST-CSL/Notch1 complexes, **C**) GST-CSL/Notch1/MAML-1 complexes, and **D**) GST-Notch1 alone. C: CSL; N: Notch; M: MAML. Cartoons representing CSL, Notch, and MAML proteins are colored green, blue, and red, respectively.

To assess the effects of added Notch and MAML-1 on the specificity of CSL for its recognized DNA target motifs, we used a microarray with 4 chambers, each containing a different preformed protein complex. We used as a negative control GST-Notch1 alone, which lacks the ability to bind DNA. Analysis of the data from the PBM chamber incubated with CSL alone revealed an 8-bp binding site consensus of YGTGGGAA (Figure 2A), which matches previous reports [12]. The comprehensive binding data also reveal a substantial amount of degeneracy at Y1, G2, G5, and A8, with more stringent constraints at T3, G4, G6, and A7.

In the same experiments, we also determined the DNA binding site preferences of CSL-Notch1 and CSL-Notch1-MAML-1 complexes and compared these results with the site preferences of CSL alone (Figure 2B, C). The DNA binding motif recognized by CSL was preserved in these complexes, and the site preferences were not detectably affected by Notch1 or MAML-1 loading. GST-Notch1, which was the negative control for these assays, did not exhibit detectable binding to the PBM (Figure 2D).

**Figure 3:**
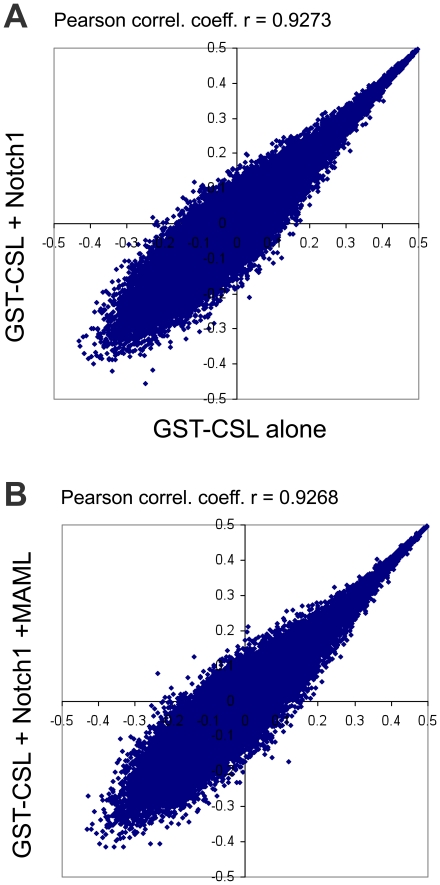
Comparison of sequence preferences for GST-CSL alone compared with GST-CSL in complexes containing Notch1 and MAML-1. Scatter plots compare PBM enrichment scores of individual 8-mers for: A) GST-CSL versus GST-CSL/Notch1 complexes, B) GST-CSL versus GST-CSL/Notch1/MAML-1 complexes. Enrichment scores were determined from the experiments shown in Figure 2.

A more comprehensive statistical analysis of the E-scores of all 32,896 ungapped 8-mers (reverse complements are merged), comparing different pairs of conditions (CSL versus Notch1-CSL, and CSL versus MAML-1-Notch1-CSL), shows that the E-scores of the different 8-mers correlate very tightly among the three conditions, providing additional support for the conclusion that the distribution of bound sites is not altered upon loading of Notch1 and MAML-1 (Figure 3).

**Figure 4:**
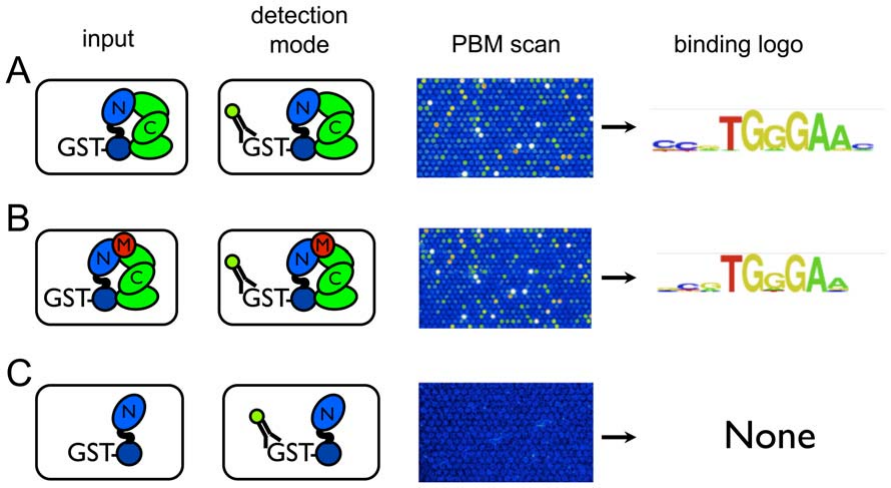
Detection of CSL-Notch1 complexes on DNA by monitoring the capture of GST-Notch-1. Columns from left to right show: experimental design, strategy for detection of immobilized complexes, PBM scans, and a DNA sequence logo representing the bound 8-mers. The protein mixtures incubated in separate chambers of the same microarray chip were as follows: A) GST-Notch1/CSL-His_6_ complexes, B) GST-Notch1/CSL-His_6_/MAML complexes, and C) GST-Notch1 alone (see methods). C: CSL; N: Notch; M: MAML. Cartoons representing CSL-His_6_, Notch, and MAML proteins are colored green, blue, and red, respectively.

To confirm that our approach detected multiprotein complexes bound to the DNA, and not merely GST-CSL that was no longer in complex with Notch1 (or MAML-1), we also assembled complexes using hexahistidine-tagged CSL, and prepared a GST-Notch1 fusion protein for use in place of unlabeled Notch1. This scheme allowed for indirect detection of CSL binding to DNA by monitoring the subsequent capture of GST-Notch1 by CSL-DNA complexes [21]. Again, the observed DNA binding site preferences closely resembled those seen upon binding of GST-CSL alone, with GST-Notch1 alone serving as the negative control (Figure 4). This experiment provides an unambiguous demonstration that it is possible to monitor the loading of multiple protein components onto DNA using PBMs.

**Figure 5:**
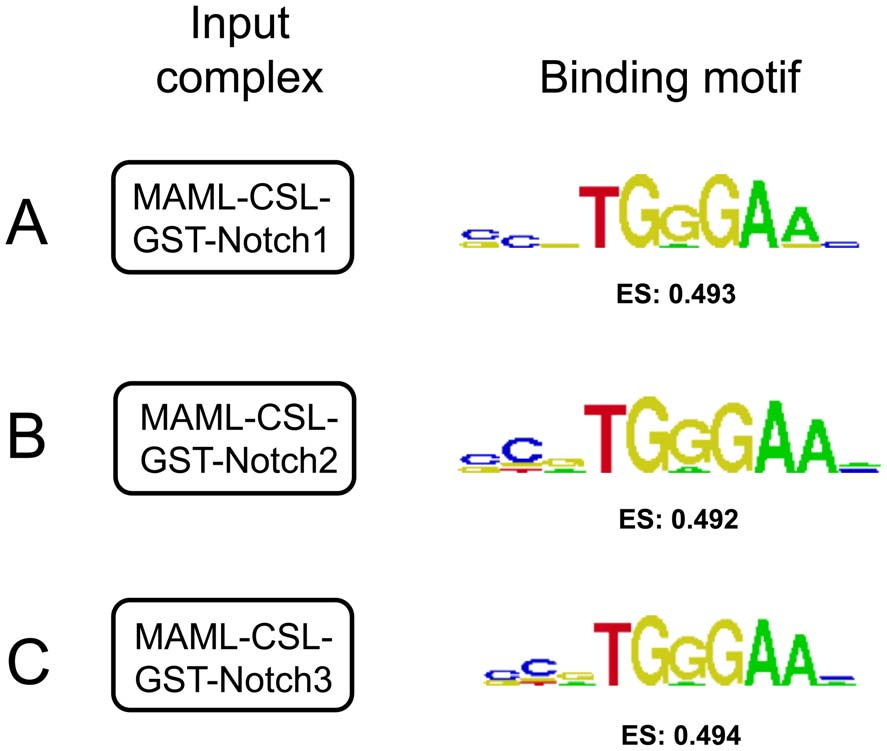
Notch receptors 1–3 do not detectably alter the DNA binding-site preferences of CSL-His_6_. DNA binding specificity motifs and 8-mer PBM enrichment scores were calculated for complexes comprised of A) CSL-His_6_ and GST-Notch1, B) CSL-His_6_ and GST-Notch2 and C) CSL-His_6_ and GST-Notch3. Motifs were derived using the Seed-and-Wobble algorithm as previously described [20], [31].

Finally, we investigated how incorporating the RAMANK domain from the four different Notch homologues (Notch1-4) into CSL-containing complexes influenced the DNA-binding properties of CSL. This experiment was performed with preassembled complexes of unlabeled CSL, GST-Notch, and MAML-1 to ensure that all detected complexes included both Notch and CSL, with the presence of MAML-1 inferred based on previous studies [7], [8], [22]. We inspected the PBM-derived motifs for potential differences among the difference complexes. We also performed an analysis of the comprehensive 8-mer PBM data to look for reproducible trends (here, 6-mers) that may be consistently favored or disfavored for binding by any of the protein complexes as compared to GST-CSl alone. The data do not reveal any detectable differences – either by examination of the PBM-derived motifs or by a comprehensive search for potential preferred 6-mers (see Methods) – in the binding specificities of CSL for DNA when it is assembled in complexes with the four different Notch receptors, nor for the different Notch complexes when compared with one another (Figure 5 and Supplementary Figure S1), leading to the conclusion that CSL DNA binding site preferences are essentially unaffected by complexation with any of the human Notch proteins (see also Supplementary Figure S2).

## Discussion

Here, we report a comprehensive study designed to uncover how DNA binding-site preferences are affected when CSL is part of a multiprotein complex. Our approach relied on the use of PBMs, a method that permits rapid, high-throughput characterization of the *in vitro* DNA binding specificities of proteins [16], [17], [18]. Universal PBM experiments covering all possible 10-mer binding sites previously have been used to identify the DNA binding preferences of over 400 eukaryotic DNA binding proteins from over 24 structural classes [20], [21], [23], [24], [25], [26], [27], [28], [29].

Here, PBM experiments performed with the Notch pathway transcription factor CSL reveal that the preferred DNA binding site for CSL is YGTGGGAA. This consensus site conforms to the published consensus determined using traditional selection methods [12]. The comprehensive, high resolution nature of the PBM data provide additional insight into the details of the DNA binding specificity of CSL: the four nucleotides underlined in the consensus sequence are nearly invariant among the bound sequences, whereas nucleotide substitutions at the other four positions are tolerated better.

The other issue addressed in these studies was whether or not the DNA site preferences of CSL vary upon complexation with Notch and Mastermind proteins. Prior studies using universal PBMs examined the DNA binding preferences of either individual proteins or unambiguous dimers [20], [21], [23], [24], [25], [26], [27], [28], [29]. To our knowledge, our study is the first to use such PBMs to investigate the potential influence of the addition of protein cofactors into multiprotein complexes on the DNA binding specificity of a transcription factor. Strikingly, there was no apparent distinction among the 8-mer binding preferences of any of the CSL-Notch or CSL-Notch-MAML-1 complexes examined in this study. These results suggest that protein-protein interactions with other transcriptional regulators and/or epigenetic mechanisms are the key events that control the distribution of genomic sites bound by the various CSL and Notch transcription complexes. Further investigation is needed to uncover how the distribution of bound sites is regulated in cells responding to Notch activation *in vivo*.

## Materials and Methods

### Protein expression and purification

GST-6×His-TEV-CSL-(9-435) was constructed by ligation independent cloning into the plasmid pET-41 Ek/LIC (Novagen) and expressed in *E. coli* Rosetta pLysS (Novagen). Cells were induced at an O.D. (600 nm) of 0.8 with 0.5 mM IPTG at room temperature, overnight. Cell pellets were resuspended in 25 mL of buffer 1 (0.5 M NaCl, 0.05 M Tris-HCl pH 8.5, 5 mM bME) and sonicated. Proteins from the cleared lysate were first affinity purified by incubating the lysates in batch with 5 mL of Ni-NTA Agarose beads (Qiagen) for 1 hour at 4°C. The Ni-NTA agarose beads were pelleted and the immobilized proteins were eluted with 250 mM imidazole. GST-CSL was further purified by binding to 10 mL of glutathione sepharose resin (GE Healthcare) pre-equilibrated with buffer 1. Beads were washed with buffer 1 and the GST fused CSL was eluted with 20 mM glutathione in buffer 1. The obtained protein sample was then concentrated and dialyzed against buffer 2 (0.5 M NaCl, 0.05 M Tris-HCl pH 8.5, 5 mM DTT). CSL-His_6_, MAML and Notch proteins were expressed and purified as previously described [8], [30].

### Protein binding microarray experiments

Universal ‘all 10-mer’ microarrays were synthesized (Agilent Technologies, AMADID # 015681) and converted to double-stranded DNA arrays by primer extension as described previously [20], [24]. Double-stranded microarrays were premoistened in PBS+0.01% (vol/vol) Triton X-100 for 5 min and blocked with 2% (wt/vol) nonfat dried milk (Sigma) in PBS for 1 h. Microarrays were then washed once with PBS+0.1% (vol/vol) Tween-20 for 5 min and once with PBS+0.01% (vol/vol) Triton X-100 for 2 min. The different protein mixtures were incubated for 30 minutes at room temperature in a 150 µl protein binding reaction containing 2% (wt/vol) milk, 51.3 ng/ml salmon testes DNA (Sigma), 0.2 mg/ml bovine serum albumin (New England Biolabs) in PBS. For the experiment reported in Figure 4 using GST-Notch1, complexes were formed at a 1∶1∶5 CSL (1.8 µM)∶Notch (1.8 µM)∶MAML (9 µM) molar ratio. For the experiments reported in Figures 2, 5, and Supplementary Figure S1, protein concentrations for each component were 0.2 µM, and complexes were formed at a 1∶1∶1 CSL∶Notch∶MAML molar ratio; experiments performed at a 1∶1∶5 molar ratio gave binding-site logos for Notch1 complexes that were not detectably distinguishable from those reported in Figure 2 (not shown). Preincubated protein binding mixtures were applied to the microarrays and incubated for 1 h at room temperature. Protein concentrations were optimized for different detection methods as listed below. Microarrays were washed once with PBS+0.5% (vol/vol) Tween-20 for 3 min, and then once with PBS+0.01% (vol/vol) Triton X-100 for 2 min. Alexa488-conjugated antibodies were diluted in PBS+2% (vol/vol) milk and applied to the microarray for 15 min (Figure 2) or 60 min (Figures 4, 5 and Supplementary Figure S1) in the dark. Finally, microarrays were washed twice with PBS+0.05% (vol/vol) Tween-20 for 2 min and once with PBS for 2 min. Washed slides were spun dry by centrifugation at 40 g for 5 min and analyzed. Alexa488-conjugated rabbit polyclonal antibody to GST (Molecular Probes, cat # A-11131) was used at a concentration of 50 µg/ml.

### Microarray analysis

Microarray analysis, data normalization, and DNA binding specificity analysis were performed as previously described [20], [31]. Briefly, all microarrays were scanned (GSI Lumonics ScanArray 5000) at three different laser power settings. Microarray TIFF images were quantified using GenePix Pro Version 6.0 software (Molecular Devices). Data from multiple scans of the same slide were combined using masliner (Micro-Array LINEar Regression) software [30]. For each spot, background-subtracted median intensities were calculated using the median local background, and the signal intensity at each spot was normalized by the corresponding relative amount of double-stranded DNA. Determination of binding preferences for all 8-mers and derivation of associated DNA binding site position weight matrices were calculated using the Universal PBM Analysis Suite and the Seed-and-Wobble motif derivation algorithm [20], [31]. The data from all experiments reported here is publicly available in the UNIPROBE database at http://the_brain.bwh.harvard.edu/uniprobe/, and are MIAME compliant for all applicable criteria.

For each PBM experiment, for each 6-mer we also averaged the E-scores of all ungapped 8-mers that contain it (typically, there are 32 such 8-mers for each 6-mer). To search for potential ‘TF-preferred’ *k*-mers [25], we then searched for 6-mers bound by GST-CSL at PBM enrichment score (E)>0.37 (bound at a 0.1% false discovery rate) and bound by a particular protein complex at E<0.32 (i.e., not bound well), and separately for 6-mers bound by a particular protein complex at E>0.37 and bound by GST-CSL at E<0.32. We repeated this analysis for each PBM experiment (Supplementary Figure S2). At these enrichment score thresholds, we did not obtain any 6-mers artifactually identified as ‘preferred’ when comparing independent, duplicate PBM experiments performed for two different protein complexes (duplicate PBM experiments for GST-Notch1+CSL+MM, and duplicate PBM experiments for GST-Notch2+CSL+MM).

## Supporting Information

Figure S1 Notch 4 does not detectably alter the DNA binding-site preferences of CSL-His_6_. DNA binding specificity motifs and 8-mer PBM enrichment scores were calculated for complexes comprised of A) CSL-His_6_ and GST-Notch2, B) CSL-His_6_ and GST-Notch4 and C) an empty chamber as a negative control. Motifs were derived using the Seed-and-Wobble algorithm as previously described [20], [31].
(PDF)

Figure S2 Heatmap of ungapped 8-mers bound in PBMs. Shown are all ungapped 8-mers bound at a PBM enrichment score of at least 0.30 in at least 1 PBM experiment in our data set. PBM experiments are clustered along the *x*-axis according to similarity in ungapped 8-mer binding profiles; ungapped 8-mers are clustered along the *y*-axis according to similarity across PBM experiments. Color bar indicates enrichment scores.
(PDF)
