# Supplementary material for: Notch and MAML-1 Complexation Do Not Detectably Alter the DNA Binding Specificity of the Transcription Factor CSL
Source: PLoS One. 2010 Nov 24;5(11):e15034. doi: 10.1371/journal.pone.0015034 (PMC2991368; doi:10.1371/journal.pone.0015034)
Supplement: Figure S1 — Notch 4 does not detectably alter the DNA binding-site preferences of CSL-His6. DNA binding specificity motifs and 8-mer PBM enrichment scores were calculated for complexes comprised of A) CSL-His6 and GST-Notch2, B) CSL-His6 and GST-Notch4 and C) an empty chamber as a negative control. Motifs were derived using the Seed-and-Wobble algorithm as previously described [20], [31]. (PDF) [file pone.0015034.s001.pdf]

**Supplementary Information for: Notch and MAML-1 complexation do not detectably alter the DNA binding specificity of the transcription factor CSL**


Cristina Del Bianco[1,2], Anastasia Vedenko[3], Sung Hee Choi[1], Michael F. Berger[3,5,6], Leila Shokri[3], Martha L. Bulyk[3-5,7], and Stephen C. Blacklow[1,4,5,7,8]

[1]Department of Biological Chemistry and Molecular Pharmacology and Department of Pathology, Harvard Medical School, Boston, MA 02115

[2]Current Address: Cibio Centro Interdipartimentale Biologia Integrata, University of Trento, Trento, IT

[3]Departments of Medicine and [4]Pathology, Harvard Medical School and Brigham and Women's Hospital, Boston, MA 02115

[5]Committee on Higher Degrees in Biophysics, Harvard University, Cambridge, MA 02138

[6]Current Address: The Broad Institute of MIT and Harvard, Cambridge, MA, 02142

[7]Division of Health Sciences & Technology, Harvard Medical School, Boston, MA 02115

[8]Dana Farber Cancer Institute, Department of Cancer Biology, Boston, MA 02115

Address correspondence to S.C.B.: sblacklow@partners.org and M.L.B. at mlbulyk@receptor.med.harvard.edu

**Figure S1. Notch 4 does not detectably alter the DNA binding-site preferences of CSL-His$_6$.** DNA binding specificity motifs and 8-mer PBM enrichment scores were calculated for complexes comprised of A) CSL-His$_6$ and GST-Notch2, B) CSL-His$_6$ and GST-Notch4 and C) an empty chamber as a negative control. Motifs were derived using the Seed-and-Wobble algorithm as previously described [20,31].

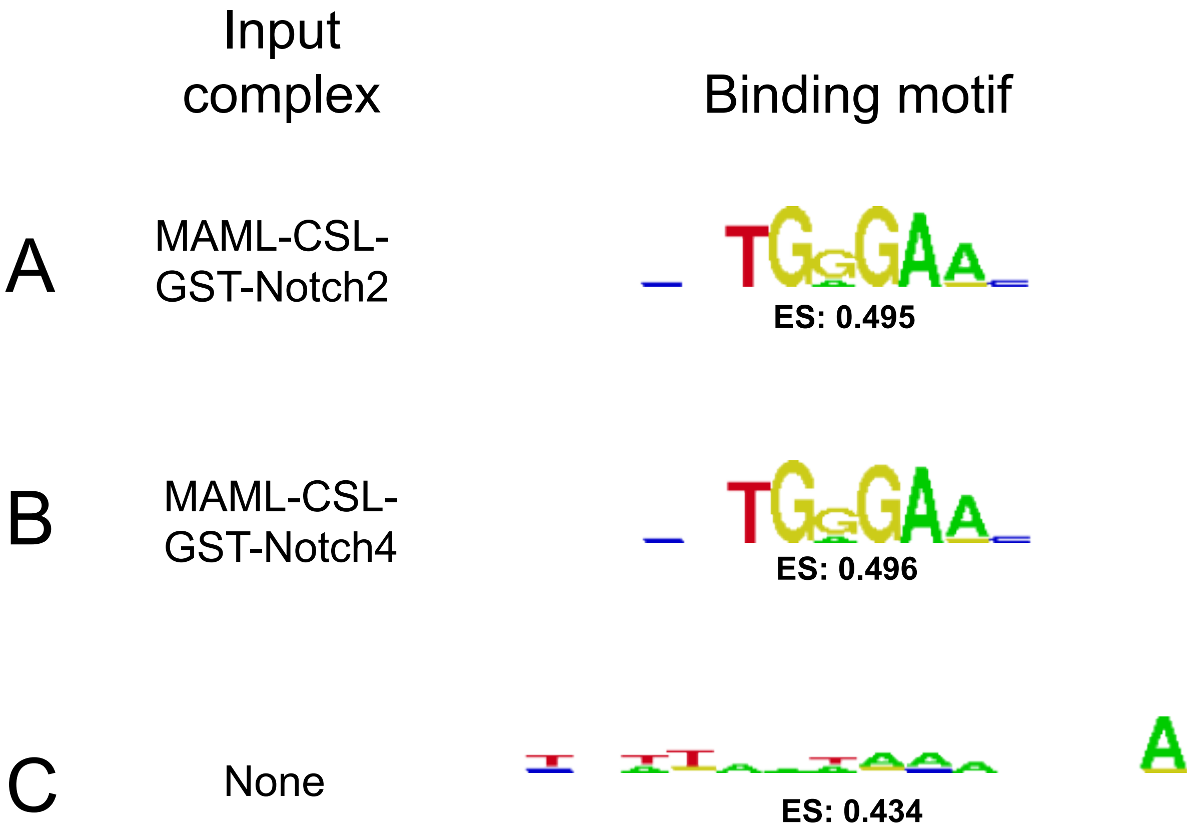
Input complex
Binding motif
A
MAML-CSL-GST-Notch2
ES: 0.495
B
MAML-CSL-GST-Notch4
ES: 0.496
C
None
ES: 0.434

Figure S1
