# Supplementary material for: Notch and MAML-1 Complexation Do Not Detectably Alter the DNA Binding Specificity of the Transcription Factor CSL
Source: PLoS One. 2010 Nov 24;5(11):e15034. doi: 10.1371/journal.pone.0015034 (PMC2991368; doi:10.1371/journal.pone.0015034)
Supplement: Figure S2 — Heatmap of ungapped 8-mers bound in PBMs. Shown are all ungapped 8-mers bound at a PBM enrichment score of at least 0.30 in at least 1 PBM experiment in our data set. PBM experiments are clustered along the x-axis according to similarity in ungapped 8-mer binding profiles; ungapped 8-mers are clustered along the y-axis according to similarity across PBM experiments. Color bar indicates enrichment scores. (PDF) [file pone.0015034.s002.pdf]

Supplementary Information for: Notch and MAML-1 complexation do not detectably alter the DNA binding specificity of the transcription factor CSL

Cristina Del Bianco[1,2], Anastasia Vedenko[3], Sung Hee Choi[1], Michael F. Berger[3,5,6], Leila Shokri[3], Martha L. Bulyk[3-5,7], and Stephen C. Blacklow[1,4,5,7,8]

[1]Department of Biological Chemistry and Molecular Pharmacology and Department of Pathology, Harvard Medical School, Boston, MA 02115

[2]Current Address: Cibio Centro Interdipartimentale Biologia Integrata, University of Trento, Trento, IT

[3]Departments of Medicine and [4]Pathology, Harvard Medical School and Brigham and Women's Hospital, Boston, MA 02115

[5]Committee on Higher Degrees in Biophysics, Harvard University, Cambridge, MA 02138

[6]Current Address: The Broad Institute of MIT and Harvard, Cambridge, MA, 02142

[7]Division of Health Sciences & Technology, Harvard Medical School, Boston, MA 02115

[8]Dana Farber Cancer Institute, Department of Cancer Biology, Boston, MA 02115

Address correspondence to S.C.B.: sblacklow@partners.org and M.L.B. at mlbulyk@receptor.med.harvard.edu

Figure S2. Heatmap of ungapped 8-mers bound in PBMs. Shown are all ungapped 8-mers bound at a PBM enrichment score of at least 0.30 in at least 1 PBM experiment in our data set. PBM experiments are clustered along the *x*-axis according to similarity in ungapped 8-mer binding profiles; ungapped 8-mers are clustered along the *y*-axis according to similarity across PBM experiments. Color bar indicates enrichment scores.

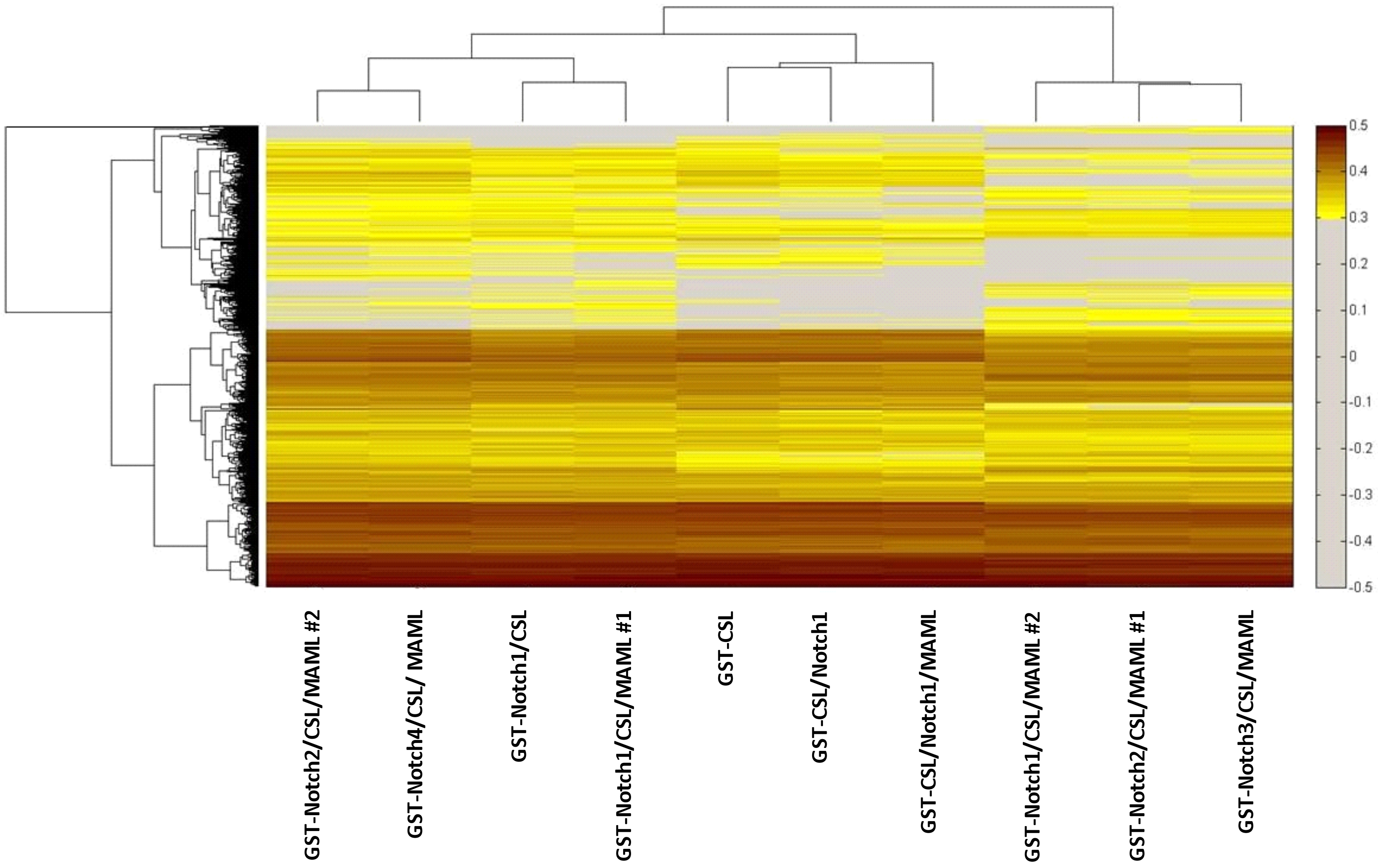
0.5
0.4
0.3
0.2
0.1
0
-0.1
-0.2
-0.3
-0.4
-0.5
GST-Notch2/CSL/MAML #2
GST-Notch4/CSL/ MAML
GST-Notch1/CSL
GST-Notch1/CSL/MAML #1
GST-CSL
GST-CSL/Notch1
GST-CSL/Notch1/MAML
GST-Notch1/CSL/MAML #2
GST-Notch2/CSL/MAML #1
GST-Notch3/CSL/MAML

Figure S2
